# Joint Analysis of Genetic Correlation, Mendelian Randomization and Colocalization Highlights the Bi-Directional Causal Association Between Hypothyroidism and Primary Biliary Cirrhosis

**DOI:** 10.3389/fgene.2021.753352

**Published:** 2021-10-04

**Authors:** Yanjun Wang, Ping Guo, Yanan Zhang, Lu Liu, Ran Yan, Zhongshang Yuan, Yongfeng Song

**Affiliations:** ^1^ Department of Biostatistics, School of Public Health, Cheeloo College of Medicine, Shandong University, Jinan, China; ^2^ Department of Endocrinology, Shandong Provincial Hospital Affiliated to Shandong University, Jinan, China; ^3^ Shandong Provincial Key Laboratory of Endocrinology and Lipid Metabolism, Jinan, China; ^4^ Institute of Endocrinology and Metabolism, Shandong Academy of Clinical Medicine, Jinan, China

**Keywords:** hypothyroidism, primary biliary cirrhosis, causal association, genome-wide association study, mendelian randomization, genetic correlation, colocalization

## Abstract

**Background:** Hypothyroidism and primary biliary cirrhosis (PBC) are often co-existed in observational epidemiological studies. However, the causal relationship between them remains unclear.

**Methods:** Genetic correlation, Mendelian randomization (MR) and colocalization analysis were combined to assess the potential causal association between hypothyroidism and PBC by using summary statistics from large-scale genome-wide association studies. Various sensitivity analyses had been conducted to assess the robustness and the consistency of the findings.

**Results:** The linkage disequilibrium score regression demonstrated significant evidence of shared genetic architecture between hypothyroidism and PBC, with the genetic correlation estimated to be 0.117 (*p* = 0.006). The OR of hypothyroidism on PBC was 1.223 (95% CI, 1.072–1.396; *p* = 2.76 × 10^−3^) in MR analysis with inverse variance weighted (IVW) method. More importantly, the results from other 7MR methods with different model assumptions, were almost identical with that of IVW, suggesting the findings were robust and convincing. On the other hand, PBC was also causally associated with hypothyroidism (OR, 1.049; 95% CI, 1.010–1.089; *p* = 0.012), and, again, similar results can also be obtained from other MR methods. Various sensitivity analyses regarding the outlier detection and leave-one-out analysis were also performed. Besides, colocalization analysis suggested that there existed shared causal variants between hypothyroidism and PBC, further highlighting the robustness of the results.

**Conclusion:** Our results suggest evidence for the bi-directional causal association between hypothyroidism and PBC, which may provide insights into the etiology of hypothyroidism and PBC as well as inform prevention and intervention strategies directed toward both diseases.

## Introduction

Primary biliary cirrhosis (PBC), known as primary biliary cholangitis, is a chronic inflammatory autoimmune disease of the liver (Carey et al., 2015). PBC is often resulted from progressive destruction of the small bile ducts of the liver, leading to cholestasis, fibrosis and eventually cirrhosis. The highest prevalence is estimated to be 40.2 per 100,000 people with the global prevalence being 14.6 per 100,000 people (Lu et al., 2018b; Trivedi and Hirschfield, 2021). PBC is much more common in women (Lu et al., 2018a), and can incur morbidity with the development of pruritus, fatigue, sicca symptoms (also known as Sjögren’s syndrome), and abdominal discomfort (European Association for the Study of the Liver, 2017). These complex clinical symptoms of PBC can be long-lasting and will result in significant damage to quality of life (Mells et al., 2013). In addition, it was estimated that up to 40% of patients had no response to standard medications for PBC (Kuiper et al., 2009; Lindor et al., 2019). Without an effective intervention, those moderately advanced or advanced PBC patients will be at high risk of liver failure and hepatocellular carcinoma and may need liver transplantation in the late stage of disease (Trivedi et al., 2016). Thus, it is necessary to identify risk factors of PBC and then benefit for the clinical prevention.

Despite vigorous efforts in the characterization of autoantibodies and bile duct histopathology, the etiology of PBC remains unclear. One common sense is that PBC is caused by genetic as well as environmental factors (Gershwin and Mackay, 2008; Carey et al., 2015). Often, the liver has an important role in thyroid hormone metabolism and the level of thyroid hormones is also important to normal hepatic function and bilirubin metabolism (Huang and Liaw, 1995). Many studies have been conducted to investigate the relationship between thyroid function and liver disease, especially between hypothyroidism and liver cirrhosis. Previous observational epidemiological studies had shown that hypothyroidism was frequently associated with PBC (Golding et al., 1973; Elta et al., 1983; Floreani and Cazzagon, 2018), and the diagnosis of hypothyroidism may precede or follow that of PBC (Elta et al., 1983). Another study found that the high prevalence of thyroid disease, especially Hashimoto’s thyroiditis, was observed in PBC patients and thyroid disease may not influence the natural history of PBC or patient survival (Floreani et al., 2017). A population-based cohort study showed that cirrhotic patients with hypothyroidism became euthyroid after thyroxine treatment, but subsequently presented some degree of liver damage (Oren et al., 2000). While other studies found that hypothyroidism was associated with an increased risk of liver fibrosis (Kim et al., 2018; Bano et al., 2020). It was not surprising to observe the inconsistent findings regarding the relationship between hypothyroidism and liver function, given that observational studies can be susceptible to measurement error, reverse causation as well as unmeasured confounders. Although many studies have confirmed that hypothyroidism has a certain association with PBC, the observational design could not allow for proving causality.

Recently, the proliferation of publicly available genome-wide association studies (GWASs) provides a rich resource of large-scale summary data that did not involve privacy and ethical issue, which has promoted the researchers to examine previously known and novel relationships among complex traits (Welter et al., 2014; Watanabe et al., 2019). Genetic correlation, Mendelian randomization (MR) and colocalization analysis are three widely-used methods to fully utilize genetic data for better understanding the genetic relationship between two traits (Burgess et al., 2018). One main advantage of these methods is that they are less sensitive to many sources of bias commonly encountered in traditional epidemiological studies. Specifically, the genetic correlation can estimate the correlation in alleles effect between two traits (e.g., hypothyroidism and PBC) across genetic variants in the whole genome (van Rheenen et al., 2019), which is symmetric in its two traits and gives no information about the direction of the correlation. While MR can assess the causal effect of an exposure (e.g., hypothyroidism) on an outcome of interest (e.g., PBC) via focusing on genome-wide significantly associated genetic variants as instrumental variables for the exposure of interest (van Rheenen et al., 2019; Kraft et al., 2020). Since genetic variants were randomly allocated from parents to offspring at conception and would not be modified, MR can be thought of a “naturally” randomized controlled trials (Haycock et al., 2016). Thus, MR is well acknowledged to be an efficient and cost-effective method to interrogate the causal relationships among health risk factors and disease outcomes (Haycock et al., 2016) as well as among molecular traits and disease outcomes (Yuan et al., 2020; Liu et al., 2021). Colocalization analysis is to examine whether two potentially related traits share common genetic causal variants in a given region (Giambartolomei et al., 2014). Unlike MR assumes that the genetic variants affect outcome only via exposure, colocalization allows the genetic variants to be associated with both traits directly. It should be noted that these three methods, though with different focus, can complement each other. Joint analysis of these three approaches can provide comprehensive investigation and better understanding for the relationship between two complex traits.

**FIGURE 1:**
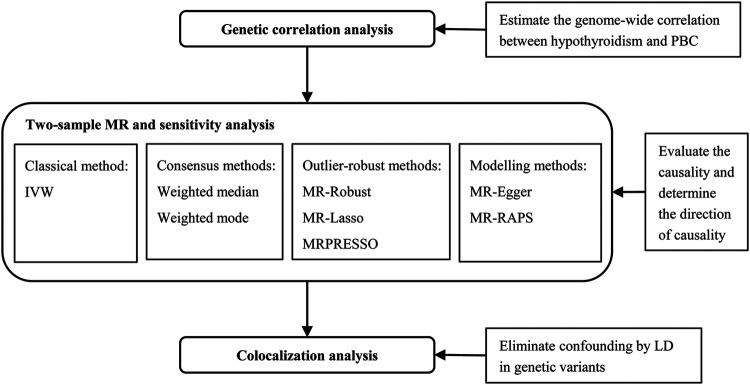
The joint analysis flowchart to comprehensively investigate the causal relationship between hypothyroidism and PBC. IVW, inverse variance weighted method; LD, linkage disequilibrium; MR, Mendelian randomization; MRPRESSO, Mendelian randomization pleiotropy residual sum and outlier; MR-RAPS, Mendelian randomization robust adjusted profile score; PBC, primary biliary cirrhosis.

At present study, we performed the joint analysis of genetic correlation, MR and colocalization to deeply demonstrate the relationship between hypothyroidism and PBC using large-scale GWAS summary statistics (Figure 1). Various sensitivity analyses had been conducted to assess the robustness and the consistency of the findings. The results can advance our understanding of the etiology of PBC and hypothyroidism, as well as provide guidance for prevention and intervention toward both diseases.

## Materials and Methods

### GWAS Data Sources

We downloaded summary statistics of hypothyroidism from the GWAS ATLAS (https://atlas.ctglab.nl/traitDB/3602), with18,740 cases and 270,568 controls from the UK Biobank (data filed 20002_1226) with 10,321,705 SNPs (Watanabe et al., 2019). Hypothyroidism was defined as self-reported history of hypothyroidism/myxoedema. The GWAS summary statistics of PBC was obtained from a genome-wide meta-analysis, which was the largest PBC GWAS to date and integrated samples from three cohorts and in total involved 2,764 cases and 10,475 controls with 1,134,141 SNPs (Cordell et al., 2015). All PBC cases fulfilled the American Association for the Study of Liver disease criteria for PBC. The details of GWAS datasets used in the analysis were briefly summarized in Table 1.

**TABLE 1:** GWAS datasets used in the present study.

Trait	Sample size	Description
Hypothyroidism	289,307 individuals of European descent, including 18,740 hypothyroidism cases and 270,567 controls	Data was from the UK Biobank. Hypothyroidism was defined as self-reported history of hypothyroidism/myxoedema
PBC	13,239 individuals of European descent, including 2,764 PBC cases and 10,475 controls	Data was from a GWAS meta-analysis, which integrated samples from three cohorts: Canadian-US cohort including 499 cases and 4,374 controls, the Italian cohort including 449 cases and 940 controls, the UK cohort including 1,816 cases and 5,161 controls. All PBC cases fulfilled the American Association for the Study of Liver disease criteria for PBC.

PBC, primary biliary cirrhosis.

### Genetic Correlation Analysis

We applied cross-trait linkage disequilibrium score regression (LDSC) to estimate the SNP heritability (*h*_2_) of hypothyroidism and PBC, and the overall genetic correlation (*r*_g_) between them (B. Bulik-Sullivan et al., 2015). LDSC was implemented by regressing the product of z statistics from two studies of traits on LD scores, which were pre-computed using 1000 Genomes European data (B. K. Bulik-Sullivan et al., 2015). We estimated the SNP heritability on the liability scale with the sample and population prevalence being 6.48 and 4.6% (Taylor et al., 2018) for hypothyroidism, and 20.88 and 0.0146% (Trivedi and Hirschfield, 2021) for PBC. The regression slope of LDSC can provide an unbiased estimate of *r*_g_. Detailed formula of cross-trait LDSC was provided in the Supplementary Methods.

### Two-Sample Mendelian Randomization

**FIGURE 2:**
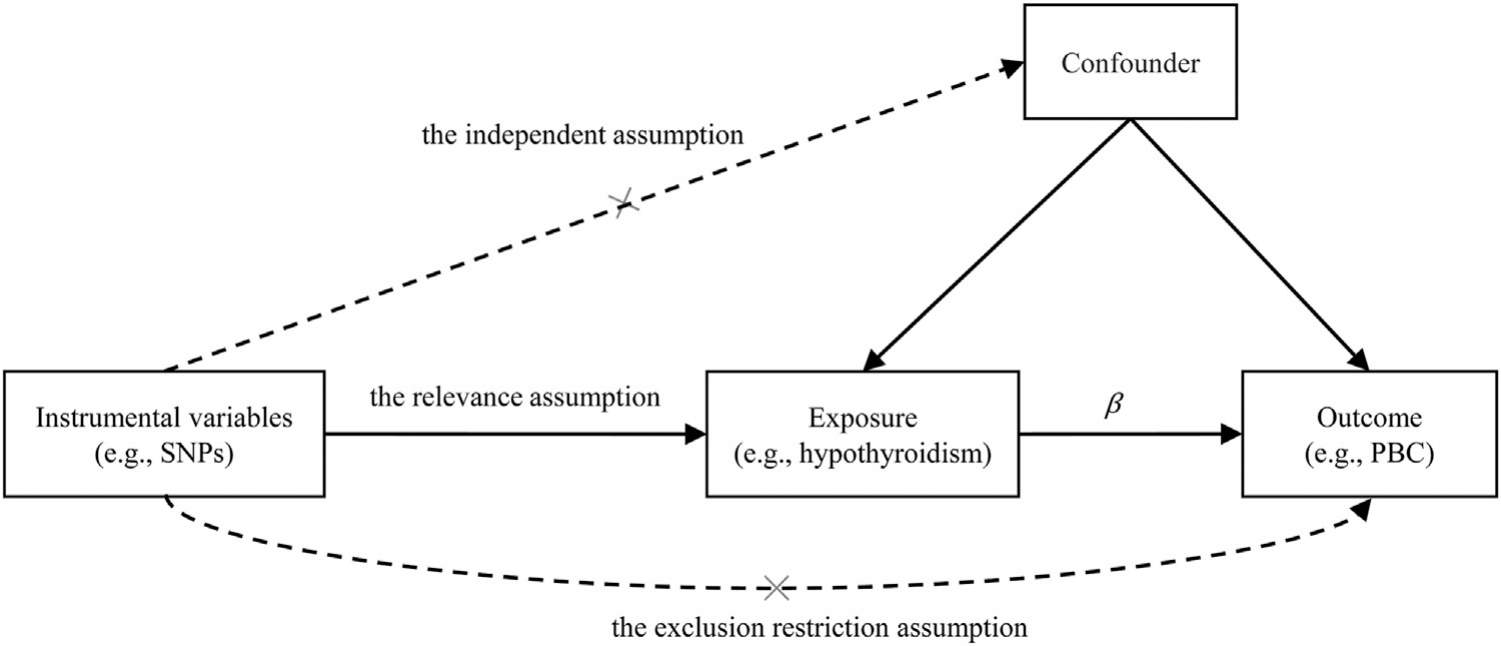
Illustrative diagram for the Mendelian randomization (MR) framework. MR analysis aims to estimate the causal effect of an exposure on the outcome *(β)* in observational studies. The plot displays the three assumptions for instrumental variable commonly required in MR analysis. Dotted lines represent possible ways that the assumptions could be violated. PBC, primary biliary cirrhosis; SNP, single nucleotide polymorphism.

We first conducted MR analysis to investigate the causal effect of hypothyroidism on PBC. Often, three assumptions should be satisfied for the genetic variant that can be served as valid instrumental variables in an MR analysis (Figure 2): 1) the genetic variant is strongly associated with the exposure (the relevance assumption); 2) the genetic variant is not associated with any potential confounding between the exposure and the outcome (the independence assumption); 3) the genetic variant only affects outcome through the exposure (the exclusion restriction assumption).

To choose the valid instrumental variables for MR analysis, we followed the previous MR study (Zeng and Zhou, 2019) with a stringent selection procedure. Specifically, we 1) selected SNPs which are strongly associated with exposure (e.g., hypothyroidism) at genome-wide significance (*p* < 5 × 10^−8^); 2) matched these significant SNPs with GWAS summary statistics of outcome (e.g., PBC) by chromosome, position and rsid, and removed those unmatched ones; 3) screened out independent SNPs using PLINK (version 1.90) (Purcell et al., 2007), based on *r* ^2^ < 0.001 or physics distance more than 10,000 kb; 4) excluded the potentially pleiotropic SNPs that are associated with outcome at the *p* value less than 0.05 after Bonferroni correction; 5) harmonized the alleles and effects between the exposure and outcome datasets. Through the rigorous screening process, there were 69 SNPs selected as instrumental variables for hypothyroidism, with detailed information shown in Supplementary Figure S1 and Supplementary Table S1. It should be noted that removing the potentially pleiotropic SNPs was a conservative strategy to sufficiently ensure the validity of MR analysis. The same MR procedure was also performed in the reverse causality analysis to investigate the causal effect of PBC on hypothyroidism. Totally, 13 SNPs were selected to serve as instrumental variables for PBC, with detailed information provided in Supplementary Figure S2 and Supplementary Table S2.

The accuracy of MR results inevitably depends on whether the selected genetic variants meet the 3 MR assumptions. The violation of the relevance assumption would lead to weak instrumental bias, we thus calculated the proportion of variance explained (PVE) by an individual SNP (Shim et al., 2015) and then computed the F statistic (Zeng and Zhou, 2019) to assess this issue, with detailed formula provided in the Supplementary Methods. In practice, it is hard to directly verify whether the independence assumption and the exclusion restriction assumption are met, thus we alternatively used multiple MR methods with different model assumptions to assess the consistency and robustness of the findings: 1) inverse variance weighted (IVW) method (Bowden et al., 2017), which requires all genetic variants to satisfy the instrumental variable assumptions; 2) weighted median method (Bowden et al., 2016a), which can provide consistent estimates when the proportion of valid instrumental variables is over 50%; 3) weighted mode-based method (Hartwig et al., 2017), which can obtain an unbiased estimate if the weights associated with the valid instrumental variables are the largest; 4) IVW method using robust regression (MR-Robust) (Burgess et al., 2016), which is robust regression-based IVW method and can obtains consistent estimates by downweighting genetic variants with potential pleiotropic effects; 5) MR-Lasso (Slob and Burgess, 2020), which uses penalization to identify the candidate instrumental SNPs; 6) MR Pleiotropy RESidual Sum and Outlier (MRPRESSO) (Verbanck et al., 2018), which first identifies horizontal pleiotropic SNPs as the outliers and then removes these outliers to infer the causal effect; 7) MR-Egger (Bowden et al., 2015), which assumes that the genetic effect on the exposure is independent of the pleiotropic effect, and it can obtain a consistent causal estimate even when all genetic variants were invalid; 8) MR Robust Adjusted Profile Score (MR-RAPS) (Zhao et al., 2020), which is robust to both systematic and idiosyncratic pleiotropy.

We further conducted the leave-one-out (LOO) analysis by removing each genetic variant out of the MR analysis in turn, to assess the influence of individual SNP on the overall causal estimate. In addition, various diagnostic plots were depicted to illustrate the MR results including scatter plot, individual SNP effect forest plot, funnel plot and LOO forest plot.

### Colocalization Analysis

Colocalization analysis was performed to assess whether hypothyroidism and PBC share common genetic causal variant in a genomic region. We implemented colocalization analysis using the commonly used Bayesian model – coloc (Giambartolomei et al., 2014), which assumes at most one association per trait in a test region and uses Approximate Bayes Factor computation to generate posterior probabilities (PP) of all possible configurations between two traits: 1) H_0_: no association with either trait; 2) H_1_: association with trait 1, not with trait 2; 3) H_2_: association with trait 2, not with trait 1; 4) H_3_: association with trait 1 and trait 2, two distinct SNPs; 5) H_4_: association with trait 1 and trait 2, one shared SNP. The PP of each configuration is respectively denoted by PP_0_, PP_1_, PP_2_, PP_3_, and PP_4_. A large PP_4_ (e.g., PP_4_ > 75%) was considered to be strong support for colocalization in the original method publication (Giambartolomei et al., 2014), which indicated a shared variant between hypothyroidism and PBC. Genomic regions were defined within 200 kb of the instrumental SNP variables, including 69 SNPs for hypothyroidism and 13 SNPs for PBC. After merging overlapping regions, we totally tested for colocalization in 79 unique regions (Supplementary Table S3). The default setting of prior probabilities in coloc were used.

## Results

### Genetic Correlation Analysis

Using LDSC, the SNP heritability on the liability scale was estimated to be 0.2085 (se = 0.0257) for hypothyroidism and 0.1527 (se = 0.0253) for PBC, respectively. We found that there existed a significant positive genetic correlation (*r*_g_ = 0.177, *p* = 0.006), implying a shared genetic architecture, between hypothyroidism and PBC. The intercept of genetic covariance was estimated to be 0.0135 (se = 0.0074, *p* = 0.0681), indicating that there was little or no sample overlap given that hypothyroidism and PBC were usually phenotypic correlated.

### Causal Effect of Hypothyroidism on PBC From MR Analysis

Totally, 69 SNPs were carefully selected as valid instrumental variables for hypothyroidism (Supplementary Table S1), which together explained about 1.79% phenotypic variance of hypothyroidism. The *F* statistics of all these instrumental variables were above 10 with an overall *F* statistic of 76.40, indicating the non-existence of weak instrument bias.

**FIGURE 3:**
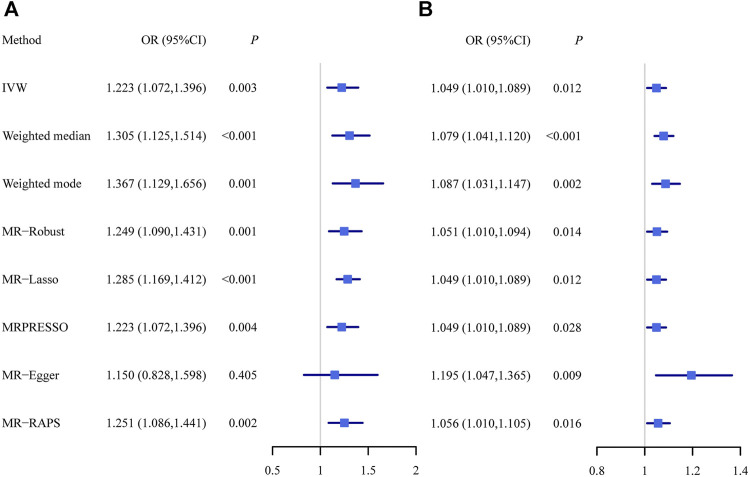
The causal effect estimates of hypothyroidism on PBC **(A)** as well as the causal effect estimates of PBC on hypothyroidism **(B)** from multiple Mendelian randomization methods with different model assumptions. IVW, inverse variance weighted method; MR, Mendelian randomization; MRPRESSO, Mendelian randomization pleiotropy residual sum and outlier; MR-RAPS, Mendelian randomization robust adjusted profile score; PBC, primary biliary cirrhosis.

Using all these selected instrumental variables, we found that hypothyroidism was positively associated with PBC. The MR estimates were reported as odds ratios (ORs) which can be interpreted as the increase of PBC risk per unit increase in log odds of hypothyroidism. The OR of hypothyroidism on PBC was estimated to be 1.223 (95% CI, 1.072–1.396; *p* = 2.76 × 10^−3^) using the random-effect IVW method, indicating that people with hypothyroidism can lead to an average of 22.3% increase in the risk of PBC and suggesting hypothyroidism may play a dangerous role in the development of PBC. The causal effect estimate of hypothyroidism on PBC from other multiple MR methods were almost identical as that from IVW (Figure 3A **)**. In particular, the ORs of hypothyroidism on PBC were estimated to be 1.305 (95% CI, 1.125–1.514; *p* = 4.45 × 10^−4^) in weighted median method, 1.367 (95% CI, 1.129–1.656; *p* = 1.36 × 10^−3^) in weighted mode-based method, 1.249 (95% CI, 1.090–1.431; *p* = 1.36 × 10^−3^) in MR-Robust, 1.285 (95% CI, 1.169–1.412; *p* = 2.08 × 10^−7^) in MR-Lasso, 1.223 (95% CI, 1.072–1.396; *p* = 3.85 × 10^−3^) in MRPRESSO, 1.150 (95% CI, 0.828–1.598; *p* = 0.405) in MR-Egger with its intercept indicating no directional pleiotropy (*p* = 0.687), and 1.251 (95% CI, 1.086–1.441; *p* = 1.89 × 10^−3^) in MR-RAPS.

**FIGURE 4:**
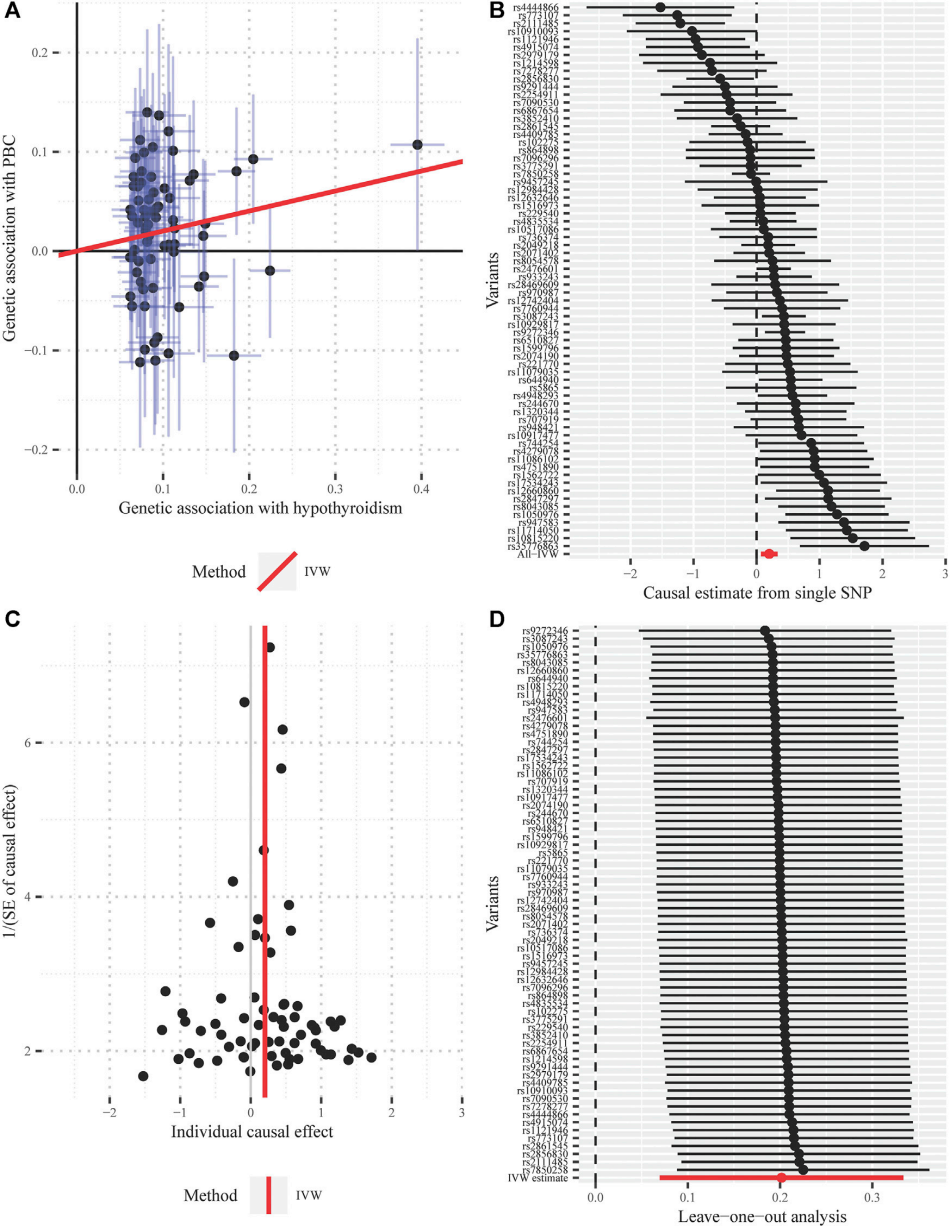
Diagnostic plots for the Mendelian randomization analysis inferring the causal effect of hypothyroidism on PBC with a total of 69 instrumental SNPs (**A)** Scatter plot between the SNP effect size estimate of hypothyroidism and the corresponding effect size estimate of PBC **(B)** Forest plot for individual causal effect estimate, with each point representing the causal effect by IVW if only using the specific SNP on the left side **(C)** Funnel plot for individual causal effect estimate **(D)** Forest plot for leave-one-out analysis, with each point denoting the causal effect by IVW after removing the specific SNP on the left side. IVW, inverse variance weighted method; PBC, primary biliary cirrhosis; SNP, single nucleotide polymorphism.

One SNP (rs2111485) was identified as a potential pleiotropic outlier at the nominal significance level of 0.05 in the MRPRESSO outlier test. However, the causal effect estimate did not change substantially after outlier correction (OR = 1.247; 95% CI, 1.098–1.417; *p* = 1.19 × 10^−3^), and MRPRESSO distortion test further suggested that there was no significant difference in the causal estimates before and after correction for outliers (*p* = 0.768). The funnel plot indicated there was no obvious outlier, strengthening the robustness of our results (Figure 4). In addition, the LOO method also suggested that no single instrument acted as a potential outlier (Figure 4).

### Causal Effect of PBC on Hypothyroidism From MR Analysis

We, following the same procedure as above, performed an MR analysis for the casual effect estimate of PBC on hypothyroidism. We identified a total of 13 SNPs as valid instrumental variables for PBC (Supplementary Table S2), which together explained about 5.43% phenotypic variance of PBC. The *F* statistics of all these instrumental variables were above 10 with an overall *F* statistic of 58.36, indicating no weak instrument bias.

In terms of random-effect IVW, the OR of PBC on hypothyroidism was estimated to be 1.049 (95% CI, 1.010–1.089; *p* = 0.012), indicating that people with PBC can lead to an average of 4.9% increase in the risk of hypothyroidism. The causal effect estimate of PBC on hypothyroidism from other MR methods were almost identical as that from IVW (Figure 3B). Specifically, it was estimated to be 1.079 (95% CI, 1.041–1.120; *p* = 4.34 × 10^−5^) in weighted median method, 1.087 (95% CI, 1.031–1.147; *p* = 2.06 × 10^−3^) in weighted mode-based method, 1.051 (95% CI, 1.010–1.094; *p* = 0.014) in MR-Robust, 1.049 (95% CI, 1.010–1.089; *p* = 0.012) in MR-Lasso, 1.049 (95% CI, 1.010–1.089; *p* = 0.028) in MRPRESSO, 1.195 (95% CI, 1.047–1.365; *p* = 8.53 × 10^−3^) in MR-Egger with its intercept indicating the possibility of directional pleiotropy (*p* = 0.047), and 1.056 (95% CI, 1.010–1.105; *p* = 0.016) in MR-RAPS.

**FIGURE 5:**
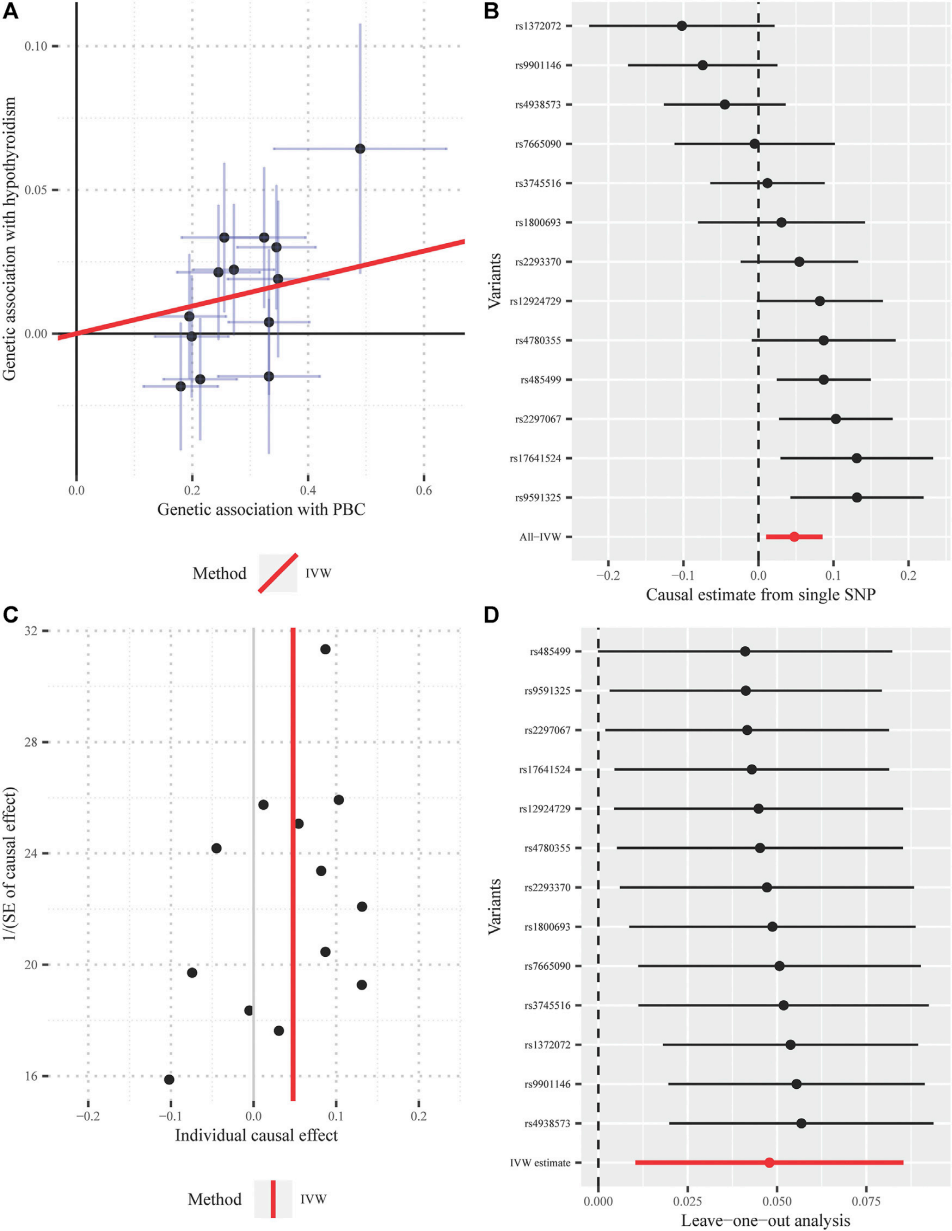
Diagnostic plots for the Mendelian randomization analysis inferring the causal effect of PBC on hypothyroidism with a total of 13 instrumental SNPs **(A)** Scatter plot between the SNP effect size estimate of PBC and the corresponding effect size estimate of hypothyroidism **(B)** Forest plot for individual causal effect estimate, with each point representing the causal effect by IVW if only using the specific SNP on the left side **(C)** Funnel plot for individual causal effect estimate **(D)** Forest plot for leave-one-out analysis, with each point denoting the causal effect by IVW after removing the specific SNP on the left side. IVW, inverse variance weighted method; PBC, primary biliary cirrhosis; SNP, single nucleotide polymorphism.

Furthermore, the MRPRESSO outlier test identified no pleiotropic SNPs at the nominal significance level of 0.05. Both the funnel plot and the LOO method also indicated the non-existence of outlier instrumental SNPs, strengthening the robustness of our results (Figure 5).

### Colocalization Analysis

Among 79 unique regions, we identified two genomic regions with PP_4_ greater than 0.75, one on chromosome two and the other on chromosome 17 (Supplementary Table S3 and Table 2), indicating that a common biological mechanism may exist between hypothyroidism and PBC. For the two regions showing evidence for colocalization, SNPs with the maximum PP_4_ were treated as the most likely shared causal variants, with their regional association plots displayed in Supplementary Figure S3.

**TABLE 2:** The shared loci between hypothyroidism and PBC identified by colocalization analysis.

Region	N_SNPs	PP.H4.abf (%)	Hypothyroidism *p*-value	Hypothyroidism SNP	PBC *p*-value	PBC SNP	Best causal
chr2:162,910,536–163,310,536	71	78.6	4.47 × 10^−16^	rs2111485	7.93 × 10^−4^	rs2111485	rs2111485
chr17:7,026,957–7,426,957	166	78.0	1.71 × 10^−10^	rs35776863	1.03 × 10^−3^	rs35776863	rs35776863

Best causal, the SNP with the highest posterior probability to be the causal variant in the genomic region; Hypothyroidism P-value and Hypothyroidism SNP, the lowest *p*-value found for hypothyroidism and the corresponding SNP; N_SNPs, the number of SNPs tested in the genomic region; PBC *p*-value and PBC SNP, the lowest *p*-value found for PBC and the corresponding SNP; PP.H4.abf, the posterior probability of hypothyroidism and PBC sharing a causal variant; Region, genomic region including chromosome and the start and stop position.

## Discussion

At present study, we jointly conducted genetic correlation, MR and colocalization analysis with large-scale GWAS summary statistics, to comprehensively investigate the causal relationship between hypothyroidism and PBC. The genetic correlation analysis showed a significant overall correlation between hypothyroidism and PBC. Generally, the observed overall association between hypothyroidism and PBC can be explained in three different ways: causality, horizontal pleiotropy and confounding by LD in alternative genetic variants (Supplementary Figure S4). Our MR analysis supported a bi-directional causal relationship between hypothyroidism and PBC, with rigorous screening for instruments to remove the horizontal pleiotropic SNPs and to minimize the impact of potential confounding factors between hypothyroidism and PBC. The consistent results from a variety of MR methods with different model assumptions guard against possible model misspecification, to ensure the reliability of the findings. Finally, colocalization analysis further ruled out the possibility of confounding due to LD between genetic variants to further strengthen the evidence of the causal relationship between hypothyroidism and PBC.

Indeed, hypothyroidism and PBC are usually co-existed (Crowe et al., 1980; Floreani et al., 2015). The bi-directional causal relationship between hypothyroidism on PBC may be biologically supported. Firstly, from the perspective of autoimmunity, the leading cause of hypothyroidism is Hashimoto’s thyroiditis, which is an autoimmune thyroiditis (Hollowell et al., 2002). Hashimoto’s thyroiditis and PBC might share a common autoimmune etiology, and there is cross-reactivity of anti-thyroid autoantibodies in the presence of autoreactive T cells or similar epithelial antigens in both the liver and the thyroid (Chalifoux et al., 2017). Apart from hypothyroidism, many studies have suggested that patients with PBC typically have an increasing incidence of other autoimmune diseases, such as, Sjögren’s syndrome, systemic sclerosis, rheumatoid arthritis, lupus, and coeliac disease (Volta et al., 2002; Narciso-Schiavon and Schiavon, 2017). Secondly, from the perspective of metabolism, thyroid hormone is critical for tissue-organ development, growth, differentiation and metabolism (Mullur et al., 2014), especially for regulating hepatic lipid, cholesterol, and glucose metabolism (Ritter et al., 2020). Hypothyroidism has been implicated in the etiology of fibrotic diseases and a number of animal studies have reported profibrotic effects of hypothyroidism (Bano et al., 2020). It is well-described that liver has a critical role in maintaining thyroid hormone homeostasis (Malik and Hodgson, 2002), and thyroid hormone binding proteins are synthesized in the liver. Approximately 80% of daily circulating triiodothyronine is produced by deiodinase enzymes from thyroxine in the liver (Perra et al., 2016). Furthermore, changes in thyroid hormone metabolism and thyroid dysfunction have been reported in chronic liver diseases (Aizawa et al., 1980; Silveira et al., 2009).

We also identified two shared causal genetic variants through colocalization analysis, including rs2111485 on chromosome two and rs35776863 on chromosome 17. The SNP rs2111485, located in the intergenic region, behaves as a *cis*-eQTL that affects IFIH1 mRNA expression (Fodil et al., 2016). As a cytoplasmic sensor of viral RNA, IFIH1, is able to activate type 1 interferon (IFN) and multiple proinflammatory cytokines and is implicated in inflammatory or autoimmune diseases. Previous study demonstrated that the enhanced expression level of type 1 IFN was observed in both the portal tract and liver parenchyma of PBC and type 1 IFN signaling may be a crucial molecular target for future treatment of PBC (Takii et al., 2005). In addition, IFIH1 has also been proved to be significantly associated with the risk of hypothyroidism in the UK Biobank (Emdin et al., 2018). These findings indicate that IFIH1 may play an important role in the relationship between hypothyroidism and PBC, and imply that a common autoimmune mechanism may exist. Another causal genetic variant, rs35776863, is important for predicting ACAP1 expression (Hoffman et al., 2017). ACAP1 is a GTPase-activating protein for ADP-ribosylation factor (Arf) 6. Previous evidence illustrate that Arf6 plays key roles in hepatic cord formation during liver development (Suzuki et al., 2006) and thyroid stimulating hormone trafficking (Lahuna et al., 2005).

To our best knowledge, this is the first comprehensive study to illuminate the bi-directional causal association between hypothyroidism and PBC by using genetic approaches based on large-scale GWAS summary statistics, where the large GWAS sample sizes provided us sufficient statistical power to detect causal association (Brion et al., 2013). Moreover, although some assumptions of instrumental variables cannot be fully tested, we have alleviated this issue by adopting rigorous procedures to select instruments and minimize the influence of potential pleiotropic SNPs. In addition, we have conducted various sensitivity analyses to show that our findings were consistent from different MR methods with different model assumptions. The results from MR-Egger are sometimes slightly different from the other methods, which may be due to the strict Egger assumption that all SNPs have the same horizontal pleiotropy effects (Burgess et al., 2018). The violations of the Egger assumption can lead to the low power of MR-Egger method (Bowden et al., 2016b). While another method, MR-RAPS (Zhao et al., 2020), relaxes such model assumption of pleiotropy and has high power to significantly detect the causal effect. It should be noted that the GWAS sample size of PBC is relatively small, and a larger PBC GWAS is certainly warranted to further verify the findings.

## Conclusion

Appropriate analysis of large-scale genetic dataset can provide some clues for biomedical research. Through the joint analysis, our study rendered strong evidence for the bi-directional causal association between hypothyroidism and PBC, which may provide insights into the etiology of hypothyroidism and PBC as well as inform prevention and intervention strategies directed toward both diseases. However, further experimental study is needed to validate the findings.

## Data Availability

All GWAS summary data analyzed during this study were publicly available and the article/Supplementary Material, further inquiries can be directed to the corresponding authors.

## References

[B1] Aizawa T. Yamada T. Tawata M. Shimizu T. Furuta S. Kiyosawa K. (1980). Thyroid Hormone Metabolism in Patients with Liver Cirrhosis, as Judged by Urinary Excretion of Triiodothyronine. J. Am. Geriatr. Soc. 28, 485–491. 10.1111/j.1532-5415.1980.tb01126.x 6253545

[B2] Bano A. Chaker L. Muka T. Mattace-Raso F. U. S. Bally L. Franco O. H. (2020). Thyroid Function and the Risk of Fibrosis of the Liver, Heart, and Lung in Humans: A Systematic Review and Meta-Analysis. Thyroid 30, 806–820. 10.1089/thy.2019.0572 31910097

[B3] Bowden J. Davey Smith G. Burgess S. (2015). Mendelian Randomization with Invalid Instruments: Effect Estimation and Bias Detection through Egger Regression. Int. J. Epidemiol. 44, 512–525. 10.1093/ije/dyv080 26050253 PMC4469799

[B4] Bowden J. Davey Smith G. Haycock P. C. Burgess S. (2016a). Consistent Estimation in Mendelian Randomization with Some Invalid Instruments Using a Weighted Median Estimator. Genet. Epidemiol. 40, 304–314. 10.1002/gepi.21965 27061298 PMC4849733

[B5] Bowden J. Del Greco M. F. Minelli C. Davey Smith G. Sheehan N. A. Thompson J. R. (2016b). Assessing the Suitability of Summary Data for Two-Sample Mendelian Randomization Analyses Using MR-Egger Regression: the Role of the I2 Statistic. Int. J. Epidemiol. 45, dyw220–1974. 10.1093/ije/dyw220 PMC5446088 27616674

[B6] Bowden J. Del Greco M F. Minelli C. Davey Smith G. Sheehan N. Thompson J. (2017). A Framework for the Investigation of Pleiotropy in Two-Sample Summary Data Mendelian Randomization. Statist. Med. 36, 1783–1802. 10.1002/sim.7221 PMC5434863 28114746

[B7] Brion M.-J. A. Shakhbazov K. Visscher P. M. (2013). Calculating Statistical Power in Mendelian Randomization Studies. Int. J. Epidemiol. 42, 1497–1501. 10.1093/ije/dyt179 24159078 PMC3807619

[B8] Bulik-Sullivan B. Finucane H. K. Finucane H. K. Anttila V. Gusev A. Day F. R. (2015). An Atlas of Genetic Correlations across Human Diseases and Traits. Nat. Genet. 47, 1236–1241. 10.1038/ng.3406 26414676 PMC4797329

[B9] Bulik-Sullivan B. K. Loh P.-R. Loh P.-R. Finucane H. K. Ripke S. Yang J. (2015). LD Score Regression Distinguishes Confounding from Polygenicity in Genome-wide Association Studies. Nat. Genet. 47, 291–295. 10.1038/ng.3211 25642630 PMC4495769

[B10] Burgess S. Bowden J. Dudbridge F. Thompson S. G. (2016). Robust Instrumental Variable Methods Using Multiple Candidate Instruments with Application to Mendelian Randomization, Available: https://arxiv.org/abs/1606.03729 .

[B11] Burgess S. Foley C. N. Zuber V. (2018). Inferring Causal Relationships between Risk Factors and Outcomes from Genome-wide Association Study Data. Annu. Rev. Genom. Hum. Genet. 19, 303–327. 10.1146/annurev-genom-083117-021731 PMC6481551 29709202

[B12] Carey E. J. Ali A. H. Lindor K. D. (2015). Primary Biliary Cirrhosis. The Lancet 386, 1565–1575. 10.1016/S0140-6736(15)00154-3 26364546

[B13] Chalifoux S. L. Konyn P. G. Choi G. Saab S. (2017). Extrahepatic Manifestations of Primary Biliary Cholangitis. Gut and Liver 11, 771–780. 10.5009/gnl16365 28292174 PMC5669592

[B14] Cordell H. J. Han Y. Han Y. Mells G. F. Li Y. Hirschfield G. M. (2015). International Genome-wide Meta-Analysis Identifies New Primary Biliary Cirrhosis Risk Loci and Targetable Pathogenic Pathways. Nat. Commun. 6, 8019. 10.1038/ncomms9019 26394269 PMC4580981

[B15] Crowe J. P. Christensen E. Butler J. Wheeler P. Doniach D. Keenan J. (1980). Primary Biliary Cirrhosis: the Prevalence of Hypothyroidism and its Relationship to Thyroid Autoantibodies and Sicca Syndrome. Gastroenterology 78, 1437–1441. 10.1016/s0016-5085(19)30497-4 6768634

[B16] Elta G. H. Sepersky R. A. Goldberg M. J. Connors C. M. Miller K. B. Kaplan M. M. (1983). Increased Incidence of Hypothyroidism in Primary Biliary Cirrhosis. Dig. Dis Sci 28, 971–975. 10.1007/BF01311724 6628157

[B17] Emdin C. A. Khera A. V. Chaffin M. Klarin D. Natarajan P. Aragam K. (2018). Analysis of Predicted Loss-Of-Function Variants in UK Biobank Identifies Variants Protective for Disease. Nat. Commun. 9, 1613. 10.1038/s41467-018-03911-8 29691411 PMC5915445

[B18] European Association for the Study of the Liver (2017). EASL Clinical Practice Guidelines: The Diagnosis and Management of Patients with Primary Biliary Cholangitis. J. Hepatol. 67, 145–172. 10.1016/j.jhep.2017.03.022 28427765

[B19] Floreani A. Cazzagon N. (2018). PBC and Related Extrahepatic Diseases. Best Pract. Res. Clin. Gastroenterol. 34-35, 49–54. 10.1016/j.bpg.2018.05.013 30343710

[B20] Floreani A. Franceschet I. Cazzagon N. Spinazzè A. Buja A. Furlan P. (2015). Extrahepatic Autoimmune Conditions Associated with Primary Biliary Cirrhosis. Clinic Rev. Allerg Immunol. 48, 192–197. 10.1007/s12016-014-8427-x 24809534

[B21] Floreani A. Mangini C. Reig A. Franceschet I. Cazzagon N. Perini L. (2017). Thyroid Dysfunction in Primary Biliary Cholangitis: A Comparative Study at Two European Centers. Am. J. Gastroenterol. 112, 114–119. 10.1038/ajg.2016.479 27779196

[B22] Fodil N. Langlais D. Gros P. (2016). Primary Immunodeficiencies and Inflammatory Disease: A Growing Genetic Intersection. Trends Immunol. 37, 126–140. 10.1016/j.it.2015.12.006 26791050 PMC4738049

[B23] Gershwin M. E. Mackay I. R. (2008). The Causes of Primary Biliary Cirrhosis: Convenient and Inconvenient Truths. Hepatology 47, 737–745. 10.1002/hep.22042 18098322

[B24] Giambartolomei C. Vukcevic D. Schadt E. E. Franke L. Hingorani A. D. Wallace C. (2014). Bayesian Test for Colocalisation between Pairs of Genetic Association Studies Using Summary Statistics. Plos Genet. 10, e1004383. 10.1371/journal.pgen.1004383 24830394 PMC4022491

[B25] Golding P. L. Smith M. Williams R. (1973). Multisystem Involvement in Chronic Liver Disease. Am. J. Med. 55, 772–782. 10.1016/0002-9343(73)90258-1 4356714

[B26] Hartwig F. P. Davey Smith G. Bowden J. (2017). Robust Inference in Summary Data Mendelian Randomization via the Zero Modal Pleiotropy assumption. Int. J. Epidemiol. 46, 1985–1998. 10.1093/ije/dyx102 29040600 PMC5837715

[B27] Haycock P. C. Burgess S. Wade K. H. Bowden J. Relton C. Davey Smith G. (2016). Best (But Oft-Forgotten) Practices: the Design, Analysis, and Interpretation of Mendelian Randomization Studies. Am. J. Clin. Nutr. 103, 965–978. 10.3945/ajcn.115.118216 26961927 PMC4807699

[B28] Hoffman J. D. Graff R. E. Emami N. C. Tai C. G. Passarelli M. N. Hu D. (2017). Cis-eQTL-based Trans-ethnic Meta-Analysis Reveals Novel Genes Associated with Breast Cancer Risk. Plos Genet. 13, e1006690. 10.1371/journal.pgen.1006690 28362817 PMC5391966

[B29] Hollowell J. G. Staehling N. W. Flanders W. D. Hannon W. H. Gunter E. W. Spencer C. A. (2002). Serum TSH, T4, and Thyroid Antibodies in the United States Population (1988 to 1994): National Health and Nutrition Examination Survey (NHANES III). J. Clin. Endocrinol. Metab. 87, 489–499. 10.1210/jcem.87.2.8182 11836274

[B30] Huang M.-J. Liaw Y.-F. (1995). Clinical Associations between Thyroid and Liver Diseases. J. Gastroenterol. Hepatol. 10, 344–350. 10.1111/j.1440-1746.1995.tb01106.x 7548816

[B31] Kim D. Kim W. Joo S. K. Bae J. M. Kim J. H. Ahmed A. (2018). Subclinical Hypothyroidism and Low-Normal Thyroid Function Are Associated with Nonalcoholic Steatohepatitis and Fibrosis. Clin. Gastroenterol. Hepatol. 16, 123–131e1. 10.1016/j.cgh.2017.08.014 28823829

[B32] Kraft P. Chen H. Lindström S. (2020). The Use of Genetic Correlation and Mendelian Randomization Studies to Increase Our Understanding of Relationships between Complex Traits. Curr. Epidemiol. Rep. 7, 104–112. 10.1007/s40471-020-00233-6 33552841 PMC7863746

[B33] Kuiper E. M. M. Hansen B. E. de Vries R. A. den Ouden-Muller J. W. den Ouden–Muller T. J. M. Haagsma E. B. (2009). Improved Prognosis of Patients with Primary Biliary Cirrhosis that Have a Biochemical Response to Ursodeoxycholic Acid. Gastroenterology 136, 1281–1287. 10.1053/j.gastro.2009.01.003 19208346

[B34] Lahuna O. Quellari M. Achard C. Nola S. Méduri G. Navarro C. (2005). Thyrotropin Receptor Trafficking Relies on the hScrib-Βpix-GIT1-ARF6 Pathway. EMBO J. 24, 1364–1374. 10.1038/sj.emboj.7600616 15775968 PMC1142541

[B35] Lindor K. D. Bowlus C. L. Boyer J. Levy C. Mayo M. (2019). Primary Biliary Cholangitis: 2018 Practice Guidance from the American Association for the Study of Liver Diseases. Hepatology 69, 394–419. 10.1002/hep.30145 30070375

[B36] Liu L. Zeng P. Xue F. Yuan Z. Zhou X. (2021). Multi-trait Transcriptome-wide Association Studies with Probabilistic Mendelian Randomization. Am. J. Hum. Genet. 108, 240–256. 10.1016/j.ajhg.2020.12.006 33434493 PMC7895847

[B37] Lu M. Li J. Haller I. V. Romanelli R. J. VanWormer J. J. Rodriguez C. V. (2018a). Factors Associated with Prevalence and Treatment of Primary Biliary Cholangitis in United States Health Systems. Clin. Gastroenterol. Hepatol. 16, 1333–1341e6. 10.1016/j.cgh.2017.10.018 29066370

[B38] Lu M. Zhou Y. Haller I. V. Romanelli R. J. VanWormer J. J. Rodriguez C. V. (2018b). Increasing Prevalence of Primary Biliary Cholangitis and Reduced Mortality with Treatment. Clin. Gastroenterol. Hepatol. 16, 1342–1350e1. 10.1016/j.cgh.2017.12.033 29277621

[B39] Malik R. Hodgson H. (2002). The Relationship between the Thyroid Gland and the Liver. QJM 95, 559–569. 10.1093/qjmed/95.9.559 12205333

[B40] Mells G. F. Pells G. Newton J. L. Bathgate A. J. Burroughs A. K. Heneghan M. A. (2013). Impact of Primary Biliary Cirrhosis on Perceived Quality of Life: the UK-PBC National Study. Hepatology 58, 273–283. 10.1002/hep.26365 23471852

[B41] Mullur R. Liu Y.-Y. Brent G. A. (2014). Thyroid Hormone Regulation of Metabolism. Physiol. Rev. 94, 355–382. 10.1152/physrev.00030.2013 24692351 PMC4044302

[B42] Narciso-Schiavon J. L. Schiavon L. L. (2017). To Screen or Not to Screen? Celiac Antibodies in Liver Diseases. Wjg 23, 776–791. 10.3748/wjg.v23.i5.776 28223722 PMC5296194

[B43] Oren R. Sikuler E. Wong F. Blendis L. M. Halpern Z. (2000). The Effects of Hypothyroidism on Liver Status of Cirrhotic Patients. J. Clin. Gastroenterol. 31, 162–163. 10.1097/00004836-200009000-00016 10993436

[B44] Perra A. Plateroti M. Columbano A. (2016). T3/TRs axis in Hepatocellular Carcinoma: New Concepts for an Old Pair. Endocr. Relat. Cancer 23, R353–R369. 10.1530/ERC-16-0152 27353037

[B45] Purcell S. Neale B. Todd-Brown K. Thomas L. Ferreira M. A. R. Bender D. (2007). PLINK: a Tool Set for Whole-Genome Association and Population-Based Linkage Analyses. Am. J. Hum. Genet. 81, 559–575. 10.1086/519795 17701901 PMC1950838

[B46] Ritter M. J. Amano I. Hollenberg A. N. (2020). Thyroid Hormone Signaling and the Liver. Hepatology 72, 742–752. 10.1002/hep.31296 32343421

[B48] Shim H. Chasman D. I. Smith J. D. Mora S. Ridker P. M. Nickerson D. A. (2015). A Multivariate Genome-wide Association Analysis of 10 LDL Subfractions, and Their Response to Statin Treatment, in 1868 Caucasians. PLoS One 10, e0120758. 10.1371/journal.pone.0120758 25898129 PMC4405269

[B49] Silveira M. G. Mendes F. D. Diehl N. N. Enders F. T. Lindor K. D. (2009). Thyroid Dysfunction in Primary Biliary Cirrhosis, Primary Sclerosing Cholangitis and Non-alcoholic Fatty Liver Disease. Liver Int. 29, 1094–1100. 10.1111/j.1478-3231.2009.02003.x 19291181

[B50] Slob E. A. W. Burgess S. (2020). A Comparison of Robust Mendelian Randomization Methods Using Summary Data. Genet. Epidemiol. 44, 313–329. 10.1002/gepi.22295 32249995 PMC7317850

[B51] Suzuki T. Kanai Y. Hara T. Sasaki J. Sasaki T. Kohara M. (2006). Crucial Role of the Small GTPase ARF6 in Hepatic Cord Formation during Liver Development. Mol. Cel Biol 26, 6149–6156. 10.1128/MCB.00298-06 PMC1592812 16880525

[B52] Takii Y. Nakamura M. Ito M. Yokoyama T. Komori A. Shimizu-Yoshida Y. (2005). Enhanced Expression of Type I Interferon and Toll-like Receptor-3 in Primary Biliary Cirrhosis. Lab. Invest. 85, 908–920. 10.1038/labinvest.3700285 15856047

[B53] Taylor P. N. Albrecht D. Scholz A. Gutierrez-Buey G. Lazarus J. H. Dayan C. M. (2018). Global Epidemiology of Hyperthyroidism and Hypothyroidism. Nat. Rev. Endocrinol. 14, 301–316. 10.1038/nrendo.2018.18 29569622

[B54] Trivedi P. J. Hirschfield G. M. (2021). Recent Advances in Clinical Practice: Epidemiology of Autoimmune Liver Diseases. Gut 70, 1989–2003. 10.1136/gutjnl-2020-322362 34266966

[B55] Trivedi P. J. Lammers W. J. van Buuren H. R. Parés A. Floreani A. Janssen H. L. A. (2016). Stratification of Hepatocellular Carcinoma Risk in Primary Biliary Cirrhosis: a Multicentre International Study. Gut 65, 321–329. 10.1136/gutjnl-2014-308351 25567117

[B56] van Rheenen W. Peyrot W. J. Schork A. J. Lee S. H. Wray N. R. (2019). Genetic Correlations of Polygenic Disease Traits: from Theory to Practice. Nat. Rev. Genet. 20, 567–581. 10.1038/s41576-019-0137-z 31171865

[B57] Verbanck M. Chen C.-Y. Neale B. Do R. (2018). Detection of Widespread Horizontal Pleiotropy in Causal Relationships Inferred from Mendelian Randomization between Complex Traits and Diseases. Nat. Genet. 50, 693–698. 10.1038/s41588-018-0099-7 29686387 PMC6083837

[B58] Volta U. Rodrigo L. Granito A. Petrolini N. Muratori P. Muratori L. (2002). Celiac Disease in Autoimmune Cholestatic Liver Disorders. Am. J. Gastroenterol. 97, 2609–2613. 10.1111/j.1572-0241.2002.06031.x 12385447

[B59] Watanabe K. Stringer S. Frei O. Umićević Mirkov M. de Leeuw C. Polderman T. J. C. (2019). A Global Overview of Pleiotropy and Genetic Architecture in Complex Traits. Nat. Genet. 51, 1339–1348. 10.1038/s41588-019-0481-0 31427789

[B60] Welter D. MacArthur J. Morales J. Burdett T. Hall P. Junkins H. (2014). The NHGRI GWAS Catalog, a Curated Resource of SNP-Trait Associations. Nucl. Acids Res. 42, D1001–D1006. 10.1093/nar/gkt1229 24316577 PMC3965119

[B61] Yuan Z. Zhu H. Zeng P. Yang S. Sun S. Yang C. (2020). Testing and Controlling for Horizontal Pleiotropy with Probabilistic Mendelian Randomization in Transcriptome-wide Association Studies. Nat. Commun. 11, 3861. 10.1038/s41467-020-17668-6 32737316 PMC7395774

[B62] Zeng P. Zhou X. (2019). Causal Effects of Blood Lipids on Amyotrophic Lateral Sclerosis: a Mendelian Randomization Study. Hum. Mol. Genet. 28, 688–697. 10.1093/hmg/ddy384 30445611 PMC6360326

[B63] Zhao Q. Wang J. Hemani G. Bowden J. Small D. S. (2020). Statistical Inference in Two-Sample Summary-Data Mendelian Randomization Using Robust Adjusted Profile Score. Ann. Stat. 48, 1742–1769. 10.1214/19-aos1866

