# Supplementary material for: Joint Analysis of Genetic Correlation, Mendelian Randomization and Colocalization Highlights the Bi-Directional Causal Association Between Hypothyroidism and Primary Biliary Cirrhosis
Source: Front Genet. 2021 Oct 4;12:753352. doi: 10.3389/fgene.2021.753352 (PMC8521021; doi:10.3389/fgene.2021.753352)
Supplement: Supplementary file 1 [file DataSheet1.DOCX]

Supplementary Material

# Supplementary Methods

## Computing the proportion of variance explained (PVE) by a given SNP and *F* statistic

For a given genetic variant (${SNP}_{i}$), we calculated the PVE following the method in (Shim et al., 2015). Specifically, PVE can be estimated by

$$\begin{aligned} {PVE}_{i}=\frac{2{\hat{\beta}_{i}}^{2}{MAF}_{i}\left( 1-{MAF}_{i} \right)}{2{\hat{\beta}_{i}}^{2}{MAF}_{i}\left( 1-{MAF}_{i} \right)+\left( se\left( \hat{\beta}_{i} \right) \right)^{2}2N_{i}{MAF}_{i}\left( 1-{MAF}_{i} \right)},\#\left( AUTONUM \backslash* Arabic \right) \end{aligned}$$

where $\hat{\beta}_{i}$ is effect size estimate, ${MAF}_{i}$ is minor allele frequency and $N_{i}$ is sample size for the ${SNP}_{i}$. The overall phenotypic variance explained by all instrumental SNP variables can be calculated by summing individual PVEs.

*F* statistic could be calculated following the method in (Zeng and Zhou, 2019). We computed *F* statistic via

$$\begin{aligned} F_{i}=\frac{{PVE}_{i}\left( N_{i}-1-k \right)}{k-k\times{PVE}_{i}},\#\left( AUTONUM \backslash* Arabic \right) \end{aligned}$$

Where ${PVE}_{i}$ is the proportion of variance explained by ${SNP}_{i}$, $N_{i}$ is the sample size for ${SNP}_{i}$ and *k* is the number of SNPs. We could calculate the overall *F* statistic based on formula (2) by using overall PVE, the number of total SNPs and total sample size.

## Cross-trait linkage disequilibrium score regression (LDSC)

Cross-trait LDSC was implemented to estimate genetic correlation between two traits only using summary statistics (Bulik-Sullivan et al., 2015). Genetic correlation analysis uses genome-wide data rather than data for only significantly associated variants, to calculate the overall average genetic association. The cross-trait LDSC equation is

$$\begin{aligned} E\left[ z_{1j}z_{2j} | l_{j} \right]=\frac{\sqrt{N_{1}N_{2}}\rho_{g}}{M}l_{j}+\frac{\rho N_{S}}{\sqrt{N_{1}N_{2}}}\#\left( AUTONUM \backslash* Arabic \right) \end{aligned}$$

where $z_{ij}$ denotes the *z* score for study *i* and SNP *j*, $l_{j}$ is the LD score, $N_{i}$ is the sample size for study *i*, $\rho_{g}$ is the genetic covariance, M is the number of SNPs in the reference panel with MAF between 5% and 50%, $N_{s}$ is the number of overlapping individuals between two studies and *ρ* is the phenotypic correlation among the $N_{s}$ overlapping individuals. We could estimate the genetic covariance $\rho_{g}$ by equation (3) and then the genetic correlation $r_{g}$ could be calculated by $r_{g}={\rho_{g}}/{\sqrt{h_{1}^{2}h_{2}^{2}}}$, where $h_{i}^{2}$ denotes the SNP heritability. It can be seen from the equation (3), sample overlap only affects the intercept (the term $\rho N_{S}/\sqrt{N_{1}N_{2}}$) from the regression and not the slope. Thus, the estimate of genetic correlation will not be biased by sample overlap. For information on how to use LDSC, you could refer to the website (<https://github.com/bulik/ldsc>).

# REFERENCE

Bulik-Sullivan, B., Finucane, H. K., Anttila, V., Gusev, A., Day, F. R., Loh, P.-R., et al. (2015). An atlas of genetic correlations across human diseases and traits. *Nat Genet* 47, 1236–1241. doi: 10.1038/ng.3406

Shim, H., Chasman, D. I., Smith, J. D., Mora, S., Ridker, P. M., Nickerson, D. A., et al. (2015). A multivariate genome-wide association analysis of 10 LDL subfractions, and their response to statin treatment, in 1868 Caucasians. *PLoS One* 10, e0120758. doi: 10.1371/journal.pone.0120758

Zeng, P., and Zhou, X. (2019). Causal effects of blood lipids on amyotrophic lateral sclerosis: a Mendelian randomization study. *Hum Mol Genet* 28, 688–697. doi: 10.1093/hmg/ddy384

# Supplementary Figures


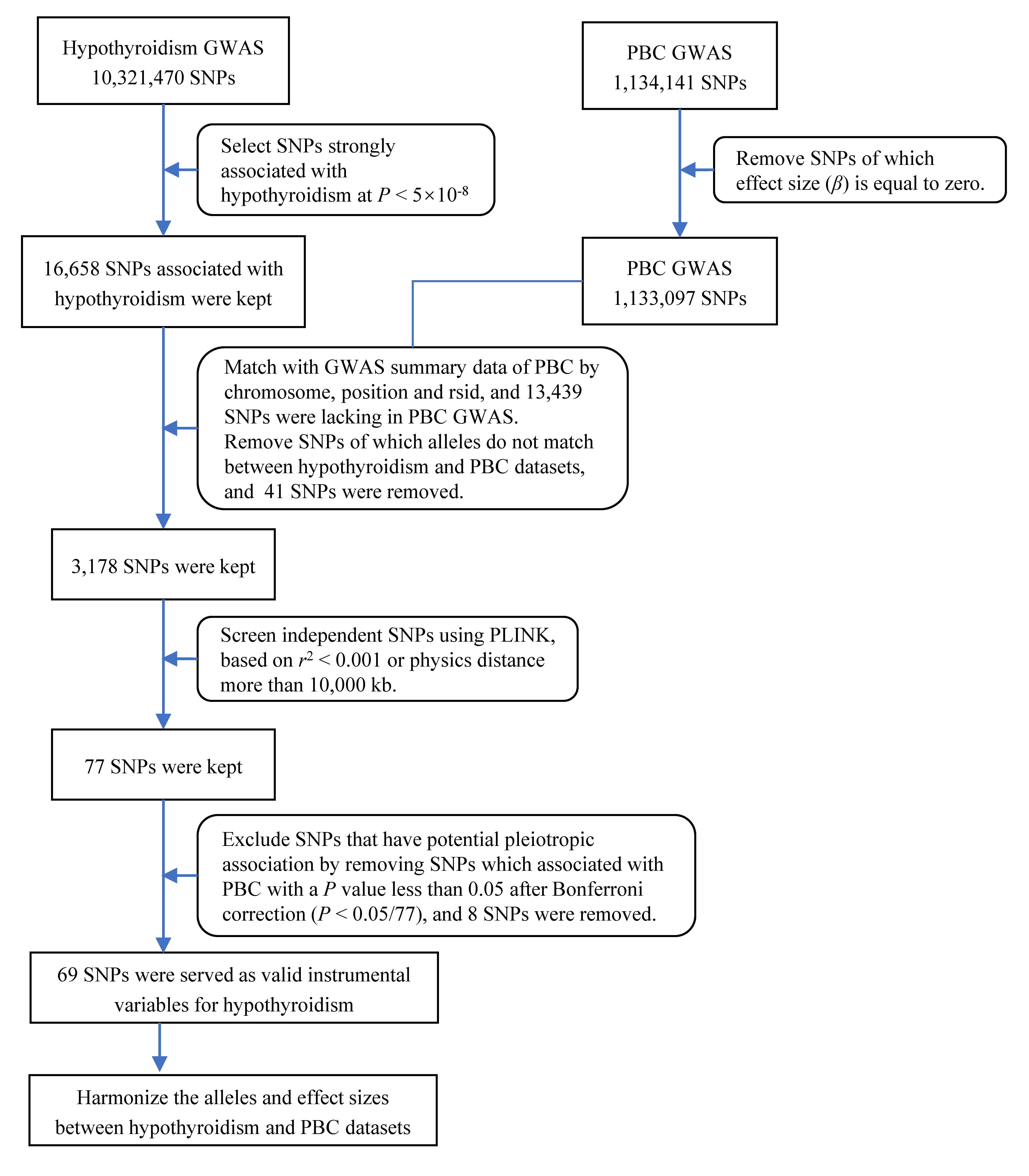


**Supplementary Figure S1.** Flowchart for the screening of instrumental SNP variables for hypothyroidism to investigate the causal effect of hypothyroidism on PBC in the MR analysis.


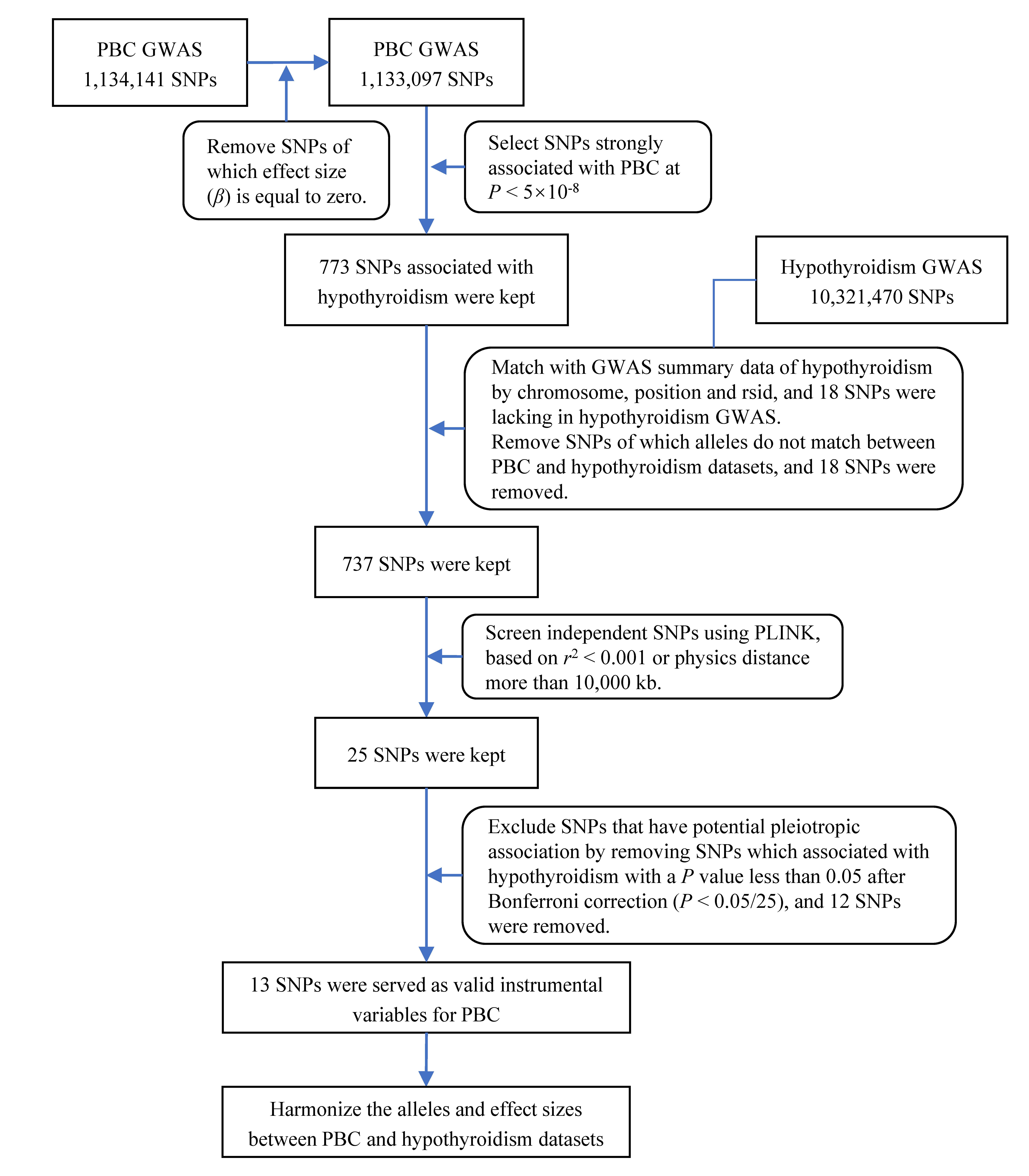


**Supplementary Figure S2.** Flowchart for the screening process of instrumental SNP variables for PBC to investigate the causal effect of PBC on hypothyroidism in the MR analysis.






(1) chr2:162910536-163310536 (2) chr17:7026957-7426957

**Supplementary Figure S3.** Regional association plots for the shared loci listed in Table 2. The labeled SNP represents the one with lowest *P*-value association for hypothyroidism (A) or PBC (B), respectively.


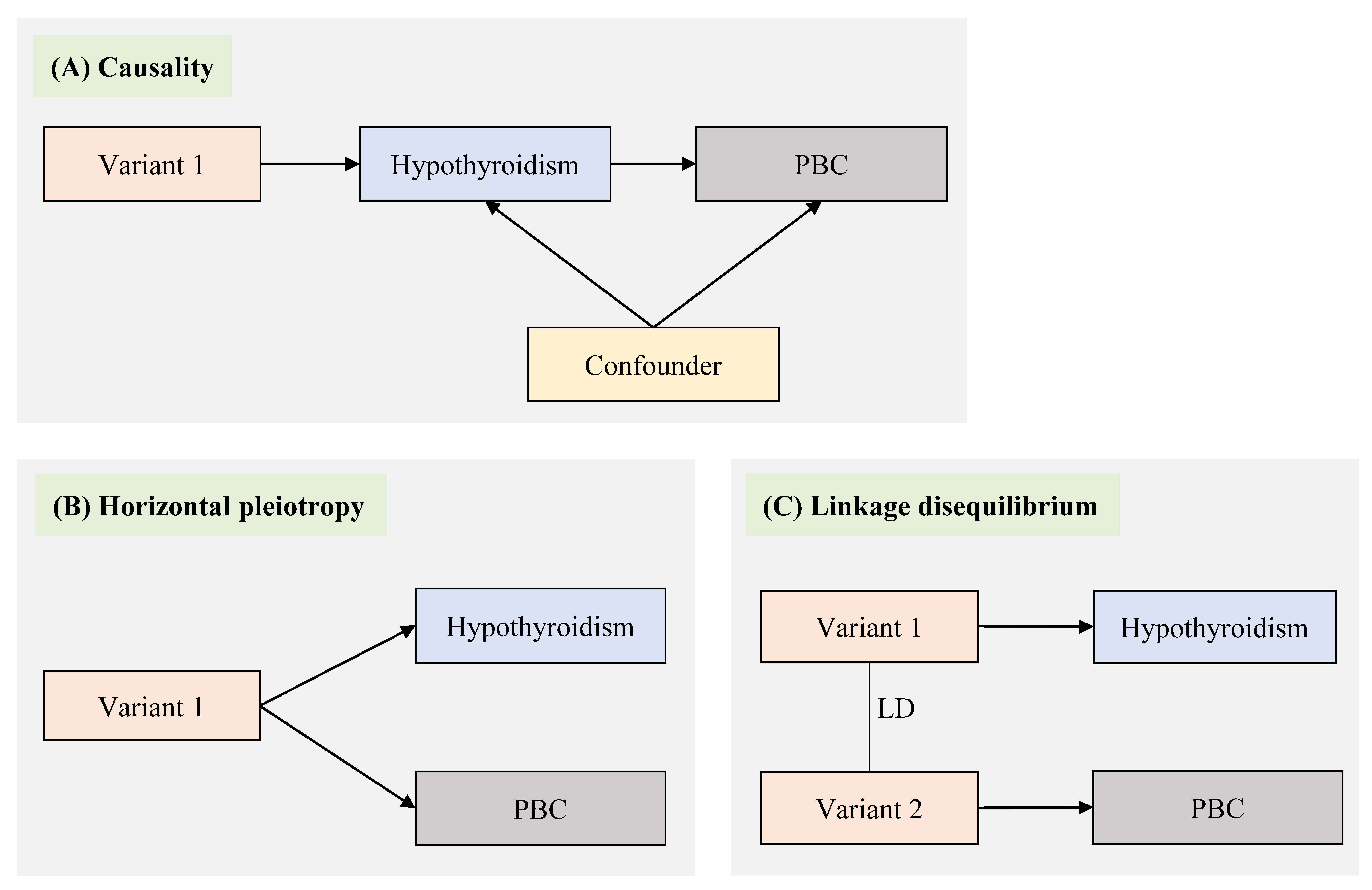


**Supplementary Figure S4.** Panel A-Causality: the genetic variant influences primary biliary cirrhosis (PBC) only via hypothyroidism. Panel B-Horizontal pleiotropy: the genetic variant influences hypothyroidism and PBC by different pathways. Panel C-Confounding by linkage disequilibrium: two correlated genetic variants influence hypothyroidism and PBC, respectively. In Mendelian randomization analysis, we removed genetic variants with potential horizontal pleiotropy, so panel B can be ruled out. Colocalization analysis could distinguish panel A from panel C.
